# Standardized assays to monitor drug sensitivity in hematologic cancers

**DOI:** 10.1038/s41420-023-01722-5

**Published:** 2023-12-01

**Authors:** Pilar Ayuda-Durán, Johanne U. Hermansen, Mariaserena Giliberto, Yanping Yin, Robert Hanes, Sandra Gordon, Heikki Kuusanmäki, Andrea M. Brodersen, Aram N. Andersen, Kjetil Taskén, Krister Wennerberg, Jorrit M. Enserink, Sigrid S. Skånland

**Affiliations:** 1 Department of Molecular Cell Biology, Institute for Cancer Research, Oslo University Hospital, Oslo, Norway; 2 Centre for Cancer Cell Reprogramming, Institute of Clinical Medicine, Faculty of Medicine, University of Oslo, Oslo, Norway; 3 Department of Cancer Immunology, Institute for Cancer Research, Oslo University Hospital, Oslo, Norway; 4 K. G. Jebsen Centre for B Cell Malignancies, Institute of Clinical Medicine, University of Oslo, Oslo, Norway; 5 Department of Haematology, Oslo University Hospital, Oslo, Norway; 6 Section for Biochemistry and Molecular Biology, Faculty of Mathematics and Natural Sciences, University of Oslo, Oslo, Norway; 7 Biotech Research and Innovation Centre (BRIC), University of Copenhagen, Copenhagen, Denmark; 8 Institute for Molecular Medicine Finland (FIMM), HiLIFE, University of Helsinki, Helsinki, Finland

**Keywords:** Haematological cancer, High-throughput screening

## Abstract

The principle of drug sensitivity testing is to expose cancer cells to a library of different drugs and measure its effects on cell viability. Recent technological advances, continuous approval of targeted therapies, and improved cell culture protocols have enhanced the precision and clinical relevance of such screens. Indeed, drug sensitivity testing has proven diagnostically valuable for patients with advanced hematologic cancers. However, different cell types behave differently in culture and therefore require optimized drug screening protocols to ensure that their ex vivo drug sensitivity accurately reflects in vivo drug responses. For example, primary chronic lymphocytic leukemia (CLL) and multiple myeloma (MM) cells require unique microenvironmental stimuli to survive in culture, while this is less the case for acute myeloid leukemia (AML) cells. Here, we present our optimized and validated protocols for culturing and drug screening of primary cells from AML, CLL, and MM patients, and a generic protocol for cell line models. We also discuss drug library designs, reproducibility, and quality controls. We envision that these protocols may serve as community guidelines for the use and interpretation of assays to monitor drug sensitivity in hematologic cancers and thus contribute to standardization. The read-outs may provide insight into tumor biology, identify or confirm treatment resistance and sensitivity in real time, and ultimately guide clinical decision-making.

## Introduction

Drug screens can identify sensitivity and resistance to anticancer drugs. The method was first introduced as the “human tumor stem-cell assay” more than four decades ago [1, 2], but was criticized as too premature for clinical implementation [3]. Since then, technological advances, development and approval of additional targeted therapies, and optimization of cell culture protocols have led to improvement, increased relevance, and validation of the method as a diagnostic tool [4–11]. In addition, the method has been used to increase our understanding of tumor biology and to stratify hematologic cancers [12–14].

Precision medicine has, in several contexts, become synonymous with genomic precision medicine. However, functional precision medicine, which guides treatment decisions based on functional tests such as drug sensitivity screens, has gained increased attention and acceptance in recent years [15]. This is showcased by a steadily increasing number of scientific publications in reputable journals, reviewed in [15–17]. A PubMed search for “functional precision medicine” retrieved 19 results in 2022, versus 5 results in 2020. To further encourage and promote research and development of functional precision medicine, the Society for Functional Precision Medicine (https://www.sfpm.io/) has been established. Their mission is to improve patient care and outcomes by facilitating the clinical implementation of functional precision medicine. Furthermore, the European Hematology Association (EHA) recently decided that the existing Specialized Working Group (SWG) “Precision Medicine” would incorporate functional precision hematology to become the new SWG “Precision Hematology”.

While neither genomic nor functional precision medicine alone has the power to identify effective treatments for all patients [16], it is likely that the integration of genomic and functional approaches can identify actionable and available drugs for a larger number of patients. It is therefore critical that the diagnostic tools are continuously assessed and validated for each application to ensure the best possible predictions and patient care. A key requirement is reproducibility between testing locations, and several clinical studies are under way to develop harmonized protocols, such as the LD-VenEx clinical trial (ClinicalTrials.gov Identifier NCT05431257), EXALT-2 (NCT04470947), and IMPRESS-Norway (NCT04817956) (Table 1). An important part of this process is the establishment of a set of community guidelines for performing drug screens.

**Table 1:** Hematologic cancers with clinically applied drug sensitivity protocols.

Cancer type	Drug sensitivity read-out	Pre-clinical/clinical application	Reference
Aggressive hematologic malignancies	Single-cell image analysis	Screen 143 patients, guide treatment of 56 patients (39%) (EXALT trial)	NCT03096821 [4]
Aggressive hematologic malignancies	Single-cell image analysis	EXALT-2 trial	NCT04470947
AML	CellTiter-Glo	Screen 187 patients, guide treatment of 37 patients (20%)	[5]
AML	Flow cytometry	VenEx trial	NCT04267081 [19]
AML	Flow cytometry	LD-VenEx trial	NCT05431257
CLL	CellTiter-Glo	Method development and application to guide treatment of an R/R patient	[8]
CLL	CellTiter-Glo	Application to guide treatment of an R/R patient	[6]
CLL	CellTiter-Glo	IMPRESS-Norway trial	NCT04817956

*AML* acute myeloid leukemia, *CLL* chronic lymphocytic leukemia, *R/R* relapsed/refractory.

Here, we present our optimized and validated protocols for drug screening of primary cells from AML, CLL, and MM patients, and of cell line models. These protocols may serve as guidelines for the use, interpretation, and further development of assays to monitor drug sensitivity in hematologic cancers.

## Drug sensitivity screening protocols

**Fig. 1:**
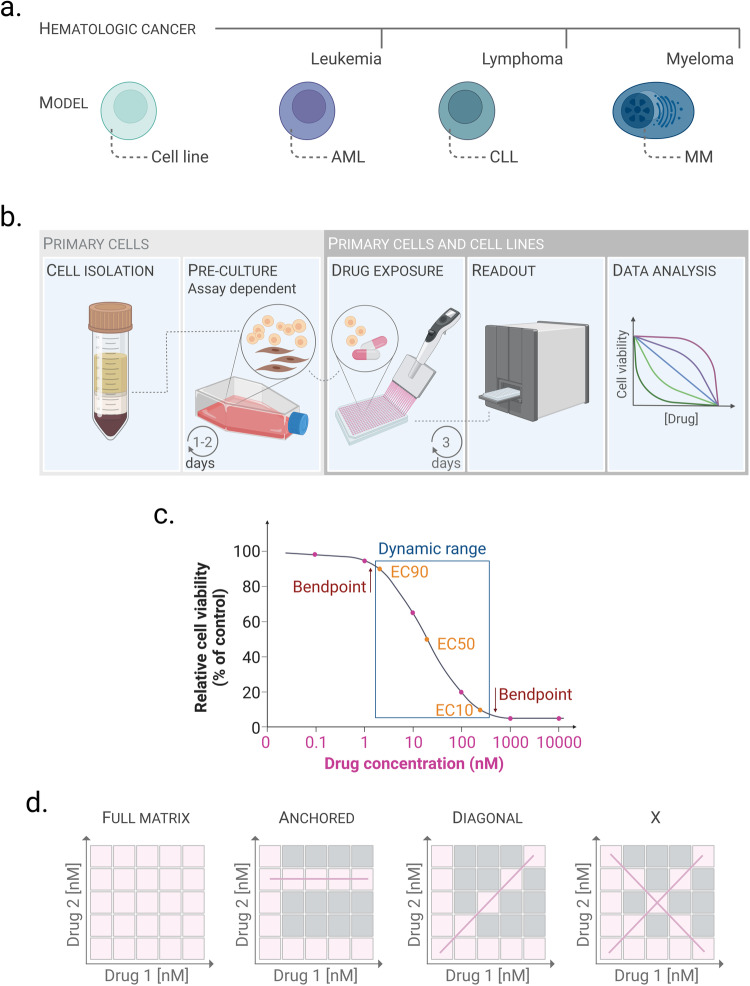
Drug sensitivity testing and drug library design. **a** Hematologic cancers are classified into three main types—leukemia, lymphoma and myeloma. We have developed drug screening protocols for cancer models that represent each of the three classes—AML, CLL and MM. Cell line models exist for these and additional diseases and can thus represent several disease types. **b** Illustration of the common principles of drug sensitivity protocols for hematologic cancers. Cell isolation and pre-culture (left panels) are specific to primary cells and are assay dependent. Primary cells or cell lines are incubated with the drug library for 3 days. Cell viability is then assessed, and the data are analyzed and presented. **c** Drawing a concentration-response curve to efficiently calculate EC50 (half maximal effective concentration) requires minimum 5 drug concentrations, and ideally 6 drug concentrations: Two before the low bend point, two after the high bend point, and at least one in the slope. From these concentration-response curves the EC50 and the dynamic range can be calculated, which is the concentration range between EC10 and EC90. The drug concentrations are usually in the nanomolar range, but must be optimized by the user for each drug and cell type. **d** Illustrations of different drug combination designs. In this example, each drug (drug 1 and drug 2) is screened at 5 different concentrations. The drugs can be combined at each included concentration (full matrix; first panel); with one of the drugs at a fixed concentration (anchored; second panel); with each drug at the same concentration (diagonal; third panel); or at the fixed-ratio x-design (x; right panel). The pink boxes indicate the tested concentrations, the gray boxes indicate the excluded drug concentrations. The pink line highlights the matrix design. The figure was created with biorender.com.

Protocols to screen for drug sensitivity have been developed for both cell line models of hematologic cancers and primary cells derived from patients. We describe protocols that have been optimized in our laboratories for drug sensitivity assessment of models that represent each class of hematologic cancer—leukemia, lymphoma and myeloma (Fig. 1a). In these protocols, the cells are exposed to drugs for 72 h. Drug exposure times may be optimized further by the user for different cell types and drug classes as sensitivity and kinetics may vary. Furthermore, primary cells are sensitive to environmental changes, and it is therefore important to control for spontaneous cell death over time to exclude non-drug induced effects on cell viability. Positive (for example, 100 µM benzethonium chloride) and negative (the drug solvent, i.e., 0.1% DMSO) controls should be included on each drug plate, which allows for measurement of the Z-prime (see the “Reproducibility and quality controls of drug screens” section) [18]. Experiments that fail this quality control should be discarded. In general, we observe that the viability of fresh primary AML cells remains around 70–80% after 48–72 h of culture, while the viability of biobanked samples may be lower ([19] and our unpublished observations). To prevent spontaneous apoptosis of primary CLL cells, they are transiently cultured with feeder cells prior to the drug exposure [8]. Primary MM cells from bulk bone marrow (BM) samples are activated with autologous BM T helper cells in the presence of IL-2 and a T-cell expansion cocktail (anti-CD3/CD28 beads) prior to drug screening [20]. Stimulation with IL-6 has also been shown to support MM survival and proliferation [21, 22]. The stimulation approaches for both CLL and MM sustain cell viability beyond the experimental window of 72 h [8, 20].

While AML and CLL patient samples are dominated by tumor cells, this is less the case for MM BM samples. The protocol for MM therefore includes a cell isolation step to avoid contamination of the result, and the pre-stimulation induces cell proliferation which allows to reach enough viable CD138^+^ MM cells to perform a drug screen [20, 23].

The common principles of drug sensitivity assays on hematologic cancers are illustrated in Fig. 1b.

### Cell lines

Any cell line, adherent or in suspension, can be analyzed with the protocol described below. Whether the assay can be performed on cells that have been in culture, or if the cells can be screened directly after thawing, needs to be determined prior to the start of the experiment. The latter may reduce variability between replicates [24, 25], but can be associated with substantial cell death resulting from the freeze-thaw cycle. Prior to the experiment it is recommended to carefully characterize the cells, establishing the optimal culturing medium, the doubling time of the cells, and the appropriate cell seeding density per well of the drug plate. This can be done by studying the cells’ growth curve over time, for example by using a live-cell imaging platform such as the Incucyte® S3 (Sartorius AG, Göttingen, Germany) which can monitor live cells in real time (Box 1). Select the cell confluency that results in optimal growth of the cells to avoid growth inhibition at the experimental end point (Box 1). The cell number needs to be optimized for the plate format to be used in the assay (i.e. 96-well, 384-well, or 1536-well plate; Box 1). Below, we describe assays performed in a 384-well plate format. Plate color (transparent, black, or white) needs to be adapted to the assay type (Box 2, 1a). Experiments should be carried out under sterile and clean conditions when indicated (see below and Box 2, 1b, c).

#### Preparation of cells (Day 1)

1. If the cells are adherent, bring them into suspension (i.e. by trypsinization). Collect the cells in a 50 mL tube and spin at 300 g for 5 min at room temperature (RT)
2. Discard the supernatant and resuspend the cells in fresh, pre-warmed medium.
3. Filter the cell suspension using a 40 µm cell strainer to assure a single-cell suspension (Box 2, 2a).
4. Count the cells using the preferred method, such as a hematocytometer or an automated cell counter.
5. Resuspend the cells in cell medium to the dilution determined based on the growth curve of the cell line (Box 1, Panel a).

#### Drug sensitivity and resistance testing (DSRT) (Day 1)

1. Transfer the cell suspension to preprinted drug plates at the appropriate volume (25 µl/well for 384-well plates) using a liquid dispenser such as the CERTUS Flex (Gwatt, Switzerland) (Fig. 1b, third panel). It is recommended to sonicate the valves of the dispenser before each use (Box 2, 1c).
2. OPTIONAL: Cover the plates with membranes that allow CO_2_ and O_2_ flow, but limit H_2_O evaporation (Box 2, 2b).
3. Leave the plates in an incubator at 5% CO_2_, 37 °C for the preferred length of time, e.g. 72 h (Box 2, 2c).

#### Measurement of cell viability with CellTiter-Glo (Day 4)

1. Equilibrate the CellTiter-Glo and the assay plates at RT for 15–30 min (Box 2, 2c). If the plates were covered with membranes (Box 2, 2b), these can be removed.
2. Add 25 μL pre-filtered CellTiter-Glo (Table 2) to each well in the 384-well plates (see Box 3 for experimental details and an alternative method).

**Table 2:** Key reagents for drug sensitivity screens.

Product	Description	Supplier	Catalogue number
Lymphoprep	Density gradient medium	PROGEN	1856-4
Lysing Buffer BD Pharm ™	RBC lysis	BD Biosciences	555899
Mononuclear Cell Medium	Medium for short maintenance of mononuclear cells	Promo Cell	C-28030
RPMI1640 Cell Medium with L-Glutamine	Medium for culturing of mononuclear cells	VWR	392-0428
384-well plate	Sterile 384 cell culture plates white, opaque bottom	Greiner Bio-one/BioNordika	781080
1536-well plate	Sterile 1536 cell culture plates white, opaque bottom	Corning Life Sciences	CLS3727
Breathe-Easy® sealing membrane	Membrane permeable to O2/CO2, to limit H2O evaporation	Merck life science (Sigma)	Z380059-1PAK
CellTiter-Glo 2.0 Assay	Luminescent cell viability assay	Promega	G9243
3. Read the luminescence with a luminometer (Fig. 1b, fourth panel).

Box 1 Optimization of cell numbers: in vitro cell proliferation Optimizing the number of cells per condition involves adjusting to the final incubation time (usually 48–96 h) and well size. The goal is to define a seeding concentration that allows the cells to grow properly and reach an end confluency of around 80–90%. If the starting density is too low, some cell lines may have problems surviving or proliferating. Too high density can confound drug effects by reaching confluence prematurely.

1. Starting number of cells per well
  This should be adapted to the type of assay plates used for the in vitro cell proliferation test. The effective growth areas for the wells in 96-, 384- and 1536- well plates are roughly 1/4 to 1/5 of the immediate larger size, and such, the standard growth volumes are 100 µL, 25 µL and 5 µL, respectively. The same fractioning may be used to convert the seeding number of cells per well between different plates. Please refer to the plate manufacturer for information about well area and recommended volume.
  If information about the doubling time and recommended seeding density is available for the cells to be used in the experiment, the starting number of cells may be approximated. Otherwise, it is recommended to use a wide concentration range. For example, for an adherent cell line seeded in a 384-well plate, a seeding number ranging from 1250 to 5000 cells/well may be a good starting point (Panel a).
2. Cell seeding
  When analyzing a suspension cell line, and in accordance with the Incucyte® manufacturer’s protocol, pre-coating the assay plate wells with a coating matrix (for example, poly-L-ornithine or fibronectin) to make the cells adhere to the well may be useful, as this prevents the cells from accumulating at the edges of the wells or displacing with movements, which can create difficulties to assess the confluency.
  Prepare the cell suspension with the highest cell concentration in growth media as a starting point for a serial dilution. Add the appropriate volume of each cell suspension to the destination wells. Seed a minimum of three wells for each cell concentration. Incubate the plates for the same time that the full experiment is planned, which will usually range from 48–96 h, and follow the evolution of the cell confluency and health during the analysis.
3. Live cell analysis with an Incucyte® S3 live-cell imaging system
  To measure the cell confluency over time one may use a live cell imaging analyzer such as the Incucyte® S3 (Sartorius AG, Göttingen, Germany), following the manufacturer’s protocol. Briefly, the device, which is placed inside a cell incubator, can take pictures using a built-in inverted microscope at the time intervals specified by the user. It is recommended to use full-well imaging which gives a full overview of the cells in the well. The instrument will collect pictures throughout the experiment. The standard interval for taking the pictures is 3–6 h, but it can be adjusted according to the user needs. Of note, select plate models that are calibrated for use with the device.
  The data are analyzed using Incucyte® Base Analysis Software, with user-defined parameters. Once the images are collected, a set of two to four representative images for different time points are selected and used to train the software to detect what is a cell, the background, and debris (Panel b, red arrows). After adjusting the parameters, the system will create a confluency mask that can be applied to all the pictures in the experiment and that will result in a confluency rate for each well and each time point. Using the software, one can simultaneously analyze the replicates for the same seeding density and draw growth curves representing confluency versus time. Panel a shows an example of an experiment performed in 384-well plate format, with four seeding concentrations (5000, 3750, 2500, and 1250 cells/well).
  Select the seeding concentration that allows the cells to be in an exponential growth phase at the end of the experiment. In the example in Panel a, the maximum confluency reached was around 95% (blue curve). High seeding density resulted in slower cell growth 24 h before the end of the incubation period and this concentration should be avoided (Panel a, blue and red curves). Low seeding density resulted in low maximum confluency (ca 40%, Panel a, purple curve), while an intermediate seeding concentration resulted in continuous cell growth and a maximum confluency of around 80% (Box 1, a, green curve). The starting concentration 2500 cells/well in a 384-well plate is therefore recommended in this example, which translates to 500 cells/well in a 1536-well plate format.
4. Visual approximation
  If advanced live cell analyzers are not available, it is possible to attempt a visual approximation of the confluency achieved in each well at the end of the experiment. The cells should have reached around 80% confluency with no signs of cell death. It is recommended to review the plates at regular intervals (for example, every 6 h).

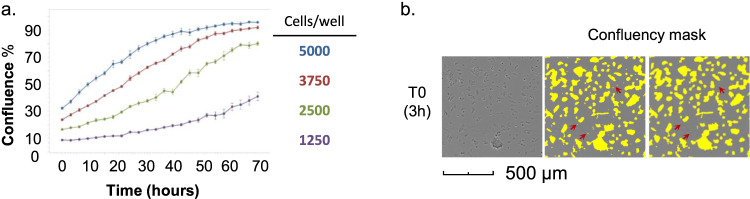

Box 2 Tips and tricks The protocol optimization in our laboratories led to the implementation of these practices, which may make a difference on experimental quality. We have classified them in different categories depending on the part of the experimental procedure where they should be used.
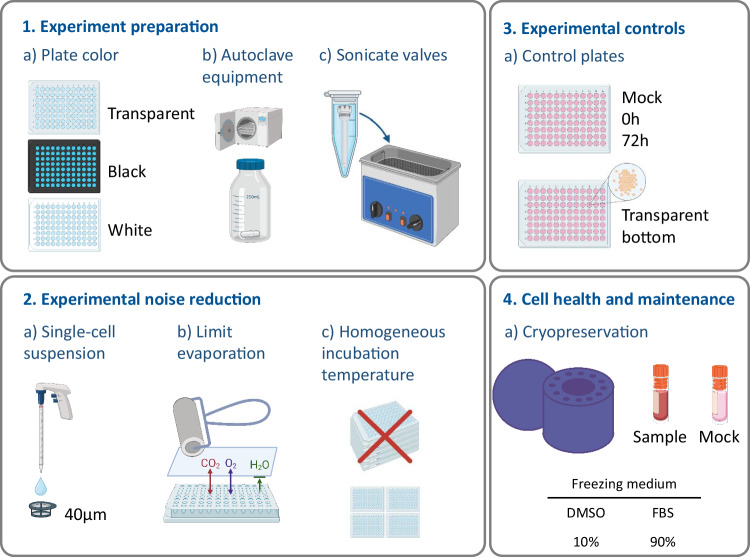

1. Experiment preparation
  - Plate color selection:
    It is important to consider the color and the transparency of the assay plates because it may affect the readout of the experiment [48].
    Luminescence: For luminescence assays the optimal color is white as it will reflect the light and provide better readings, but black is also suitable. The walls should be opaque to prevent bleeding of signal between wells. If reading the signals top-down, it is recommended to use opaque-bottom plates. This will limit the light bleeding to neighboring wells
    Fluorescence: For fluorescence assays the optimal color is black. If the assay multiplexes fluorescence and luminescence signals, gray plates can be used which compromise both assays
    Microscopy: If the readout involves microscopy, optical plates with high-quality transparent bottom should be used. If the readout involves fluorescent signals in high content imaging the use of plates with black walls is recommended
    Flow cytometry: To fully sample the well, plates with conical or round bottom wells are preferred
  - Autoclaving the material:
    The containers for the cell suspension need to be prepared in advance of the experiment. If using 50 mL tubes or syringes, they should be sterile. For bigger volumes use autoclaved bottles with a magnet stirrer, that will help to keep the cells in suspension during the assay procedure. It is useful to add the magnet stirrer to the bottle before autoclaving to have a sterile kit ready for the experiment
  - Sonicating valves:
    The valves used for cell and reagent dispensing by the liquid dispenser (e.g. CERTUS Flex, Watt, Switzerland) can get clogged, even if using a strict cleaning protocol. Sonicating the valves in an ultrasonic bath helps breaking aggregates inside the canal, improving the effectiveness of the cleaning process. Fill a 1.5 mL tube with distilled sterile water, add the valve, and introduce the tube in the ultrasonic bath. Sonicate for 15 min and clean. Repeat the procedure if necessary
2. Experimental noise reduction
  - Single cell suspension:
    When working with adherent cells or suspension cells which tend to form aggregates, the use of a 40 µm cell strainer to filter the cell suspension will help to obtain a single-cell suspension
  - Limit evaporation:
    Since the outer wells in the assay plates are more prone to evaporation of the cell medium, it is recommended to avoid using them for experimental purposes. The outer wells should be filled with medium to protect the inner wells from evaporation.
    The use of Breathe-Easy® sealing membranes (Table 2) allows the CO_2_ and O_2_ to flow, but limits H_2_O evaporation. To paste them remove the first layer (paper) and place on the open plate avoiding wrinkles. Make sure all the wells are covered by the membrane. Use a roller to properly paste the membrane to the plate. Remember to remove the second layer of plastic. Cover with the plate lid
  - Homogeneous incubation temperature:
    Both for the cell growth and the temperature equilibration before reading the cell viability the plates should be spread in a one-plate layer, never stacked on top of each other. This will assure a homogeneous distribution of the temperature, leading to less variation in the data
3. Experimental controls
  - It is recommended to include the following control plates in the experiment:
    Mock plate, if the liquid handling device is prone to start-effect (changes in cell numbers due to errors at the beginning of the procedure)
    Two undrugged plates:
      Initial plate (0 h): Reading this plate right after dispensing will provide an overview of the quality of the dispensing. Occasionally it will show experimental problems, leading to the discard of the assay
      Final incubation plate (72 h): The readout from this plate will give an indication about the variation/noise of the data over time, as a result of non-uniform cell growth
    An assay plate with transparent bottom can be included to monitor the cells and check for contaminations
4. Cell health and maintenance
  - Cryopreservation:
    If using Corning® CoolCell® containers, it is recommended to fill empty spaces with mock tubes filled with freezing medium to assure an even decrease in temperature during freezing
    Premixing the DMSO in the freezing media and cooling the solution to 4 °C prior to use is preferred. This prevents cell damage due to the exothermic reaction of DMSO addition to an aqueous solution. Preparing the freezing media in advance also prevents damage from high local concentrations of DMSO prior to the suspension being fully mixed
  - Cell Thawing:
    After thawing the cells, DNAse treatment is recommended to remove cell clumps that may form due to cell death and release of sticky DNA. Thaw the cells in pre-warmed media containing 50U of TurboNuclease (Sigma-Aldrich, St. Louis, MO, USA). There should be no dropwise addition of the warm media but rather a direct addition of the full amount (10 mL for every 1 mL of frozen cell suspension). The sample is spun (500 g for 10 min) and resuspended in warm media (5 mL) with TurboNuclease [49–52]. If the pellet does not fully dissolve, incubate at 37 °C for 5–30 min until the pellet is dissolved. The cells are spun and fresh warm media, without TurboNuclease, is added. The cells in suspension are then counted, spun, and resuspended in the appropriate media

Box 3 Measurement of cell viability with CellTiter-Glo and Flow Cytometry CellTiter-Glo viability assay detects metabolically active cells by lysing the cells and using the released ATP for an enzymatic reaction which produces luminescence. The fact that it involves an enzymatic reaction makes it sensitive to temperature changes.

1. Place the CellTiter-Glo® or CellTiter-Glo® 2.0 (Promega, WI, USA; CTG from now) at RT until the temperature is stable
2. Plate preparation:
  - Place the plates in the hood or on a table for 15–30 min until the medium reaches room temperature
  - If using Breathe-Easy® sealing membranes to prevent evaporation, these can be removed while the plate temperature equilibrates. Be careful as the contents of the wells can be spilled if the movements are sudden. Be sure to remove the whole membrane and eventually clean the plates with ethanol-soaked tissue to remove possible glue residues left by the membranes (this ethanol swiping step is necessary only if a stacker is used for automated plate handling in the next steps)
  - Add pre-filtered CTG using the liquid handling device of preference. To reduce costs, CTG can be diluted without substantial difference in the final results. For instance, CTG commercial stock can be diluted 1:1 in PBS and added at the same volume as the sample, or undiluted CTG can be used 1:2 with sample volume (for example, for 25 μL of sample volume, add 25 μL of 1:1 diluted CTG or 12.5 μL of undiluted CTG). The final dilution of sample volume:CTG will be 1:0.5
  - Wait at least 10 min and read the emitted luminescence in a luminometer such as the Envision Multimode reader (PerkinElmer, Waltham, MA, USA).

Flow cytometry assays have the advantage of supplying the user with not only the live/dead cell numbers for each treatment, but also the possibility to determine what cell types have been affected by the drug treatments. Screening flow cytometry assays are typically performed in “no-wash” formats where dyes and antibodies have been titrated to allow for the detection of cellular labeling without the need of washing the cells after staining. This prevents cell loss during wash steps, which could otherwise introduce well-to-well variability in detected cell numbers. High throughput flow cytometers, which sample cells in a continuous mode, allow for rapid reading of 96- and 384-well plates.

For the VenEx [19] and LD-VenEx (NCT05431257) clinical trials, a panel of antibodies identifying the blast population (CD34, CD45, CD117) from more differentiated leukemic cells and lymphocytes is used. The panel includes antibodies detecting cell surface markers CD45, CD34, CD38, CD117, CD11b, CD14 and CD64 as well as the apoptosis and live/dead stains Annexin V and 7-AAD. The assays are performed in 96-well pre-drugged plates after a 48 h incubation post seeding the cells onto the drugs.

1. Preparing the antibody staining solutions
  - Prepare the full stain antibody master mix as well as staining FMO (fluorescence minus one) control antibody mixes in an appropriate medium (either the culture medium with 10% FBS or PBS with 1% BSA).
2. Staining the sample
  - Centrifuge the plate containing the cells (preferably a V-bottom plate) at 500 g for 5 min and discard the supernatant by flipping the plate. Add 25 µL of the antibody master mix or FMO to the appropriate wells and incubate covered for 25 min at RT. During this time prepare the viability staining of Annexin V and 7-AAD in the binding buffer.
  - Spin the plate and remove the media. Add 25 µL of the viability staining mix and incubate covered at RT for 10 min.
  - The plate is now ready to run. An iQue Screener Plus (Sartorius) or similar high throughput flow cytometer is used to capture all the events in each well. If a non-high throughput-type flow cytometer is used, the final amount of staining solution may have to be increased or buffer added to account for dead volume requirements of the instrument.
  - Use the flow cytometer-provided software (Forecyt for iQue instruments) to complete the gating to identify live vs. dead cells as well as different leukemic and healthy cell populations. Export a data table (in the form of a comma separated value (csv) or Excel file) containing the number of events in the relevant gated cell populations for each well for downstream standard dose response analyses.
  - A detailed description of the gating strategy used to identify blasts and calculate concentration response curves has previously been reported [19]

A comparison of CTG and flow cytometry-based drug screening approaches showed a Spearman’s correlation coefficient of 0.75 (*p* < 0.0001) between the drug sensitivity scores for seven drugs in blasts from 24 AML patient samples with clinical blast count >50% [53].

### Primary AML cells

AML cells can be isolated from BM or peripheral blood (PB) and analyzed fresh or after storage in liquid nitrogen [26, 27]. A change in cell composition of the sample after cryopreservation has been reported, with a reduction of CD117^+^ cells, granulocytes, and CD45^-^ cells (erythropoietic cells) [27]. Overall, drug responses are highly correlated between fresh and frozen samples, with some exceptions [26, 27]. It is important to note that significant changes in the cell composition after thawing, such as a large reduction of granulocytic cells, can impact the drug sensitivity measurement when assessing bulk sample sensitivity. It is therefore recommended to specify the state of the samples when reporting the results. Cell culturing conditions may also impact drug screening results. See Box 4 for considerations to be made regarding culturing media.

The below protocol for drug sensitivity screening of BM or PB mononuclear cells (MCs) from AML patient samples was adapted from a previous report [9].

#### Isolation of mononuclear cells (MCs) from AML patient samples

1. Dilute the blood 1:1 with phosphate-buffered saline (PBS).
2. Prepare a density gradient medium according to the manufacturer’s instructions. As an example, if using Lymphoprep (Table 2), prepare a set of 15 mL tubes adding 3 mL of RT Lymphoprep to each tube.
3. Carefully add up to 5 mL of diluted PB or BM on top of the density gradient.
4. Centrifuge the tubes at 500 g for 20 min without breaks. The MCs will now be visible as a white layer on top of the density gradient medium (Fig. 1b, left panel).
5. Use a Pasteur pipette to transfer the MCs into a new 50 mL tube. Wash the cells twice with PBS.
6. Centrifuge the cells at 500 g for 5 min with the breaks on.
7. NOTE: In certain AML samples, the presence of erythroid cell contamination may be observed following density gradient centrifugation. Therefore, red blood cell (RBC) lysis is recommended.
  Discard the supernatant and resuspend the pellet in 2 mL 1 x RBC lysis buffer (Table 2) for 5 min at RT.
8. Stop the reaction by diluting the cell solution with 25 mL PBS.
9. Filter the cells with a 40 µm cell strainer (Box 2, 2a).
10. Count the cells using the preferred method.
11. Centrifuge at 300 g for 15 min.
12. Discard the supernatant and proceed to “Cryopreservation of MCs from AML patient samples” or “DSRT of AML patient samples (Day 1)”.

#### Cryopreservation of MCs from AML patient samples

Resuspend the cells in fetal bovine serum (FBS) supplemented with 10% DMSO, aliquot, and store in liquid nitrogen (Box 2, 4a)

#### DSRT of AML patient samples (Day 1)

MCs in AML drug screens are used at a standard count of 10,000 cells/well in a final volume of 25 μL for 384-well plates. The final volume of the cell suspension should be adapted to the total number of drug plates in the experiment. If the number of available cells is limited, the counts may be adapted. Preferably, the counts should be above 1000 cells/well, although reproducible results have been observed with as little as 300 cells/well (our unpublished observation).

##### Fresh cells

Resuspend the cells in mononuclear cell medium (MCM) to a final concentration of 4.0 × 10^5^ cells/mL. This will allow for 10,000 cells/well in a 25 μL/well volume.

##### Cryopreserved cells

Cryopreserved AML cells may show up to 40–50% viability loss due to the procedure. Calculate the final number of cells needed for the experiment and thaw twice the amount if available (Box 2, 4b). Place the cells in fresh MCM and recover overnight in an incubator at 5% CO_2_, 37 °C. Resuspend the cells in MCM to a final concentration of 4.0 × 10^5^ cells/mL. This will allow for 10,000 cells/well in a 25 μL/well volume.

1. Transfer the cell suspension to preprinted drug plates at the appropriate volume using a liquid dispenser such as the CERTUS Flex (Fig. 1b, third panel). It is recommended to sonicate the valves of the dispenser before each use (Box 2, 1c).
2. OPTIONAL: Cover the plates with membranes that allow CO_2_ and O_2_ flow, but limit H_2_O evaporation (Box 2, 2b).
3. Leave the plates in an incubator at 5% CO_2_, 37 °C for the preferred length of time, e.g., 72 h (Box 2, 2c).

#### Measurement of cell viability with CellTiter-Glo (Day 4)

1. Equilibrate the CellTiter-Glo and the assay plates at RT for 15–30 min (Box 2, 2c). If the plates were covered with membranes (Box 2, 2b), these should be removed.
2. Add 25 μL pre-filtered CellTiter-Glo (Table 2) to each well in the 384-well plates (see Box 3 for experimental details and an alternative method).
3. Read the luminescence with a luminometer (Fig. 1b, fourth panel).

Box 4 AML culturing conditions AML cells are commonly grown in RPMI or IMDM medium supplemented with 10% FBS, which can sustain cell viability for a few days. Similarly, the Mononuclear cell medium (MCM) is suitable for short-term assays. However, blasts exhibit limited proliferation and viability in these conditions.

To enhance cell viability, cytokines can be utilized. Some of these cytokines include FLT3L, SCF, TPO, G-CSF, GM-CSF, IL-3, IL-6, and IL-1B [54, 55]. A common approach to improve cell viability and to stimulate blast proliferation is to use serum-free medium, such as StemSpan SFEM II, supplemented with the selected cytokines [56]. In addition, conditioned medium derived from HS-5 human stromal fibroblast cell line has been employed to enhance blast survival [57].

Importantly, culturing conditions can significantly influence drug screening results. For example, in cytokine-poor medium (such as RPMI or MCM), the BCL-2 inhibitor venetoclax and FLT3 inhibitors show increased efficacy, while their efficacy is dramatically reduced in cytokine-containing medium [19, 58]. It is important to consider the variations introduced by culturing conditions in drug screening experiments.

### Primary CLL cells

CLL cells can be isolated from PB and analyzed fresh or after storage in liquid nitrogen. Cryopreservation of primary B cells from healthy blood donors and CLL patients has been shown to not affect cell signaling [28], supporting the use of biobanked samples for functional assays. This makes it possible to analyze historical samples, to perform several assays over time on samples collected from the same patient, and to store samples for later use.

The below protocols for isolation, cryopreservation, and drug sensitivity screening of peripheral blood mononuclear cells (PBMCs) from CLL patient samples were adapted from previous reports [8, 29, 30].

#### Isolation and cryopreservation of PBMCs from CLL patient samples

NOTE: Blood samples procured from CLL patients should be processed the same day.

1. Dilute the blood 1:1 with PBS and transfer the solution to 50 mL tubes (not more than 30 mL/tube).
2. Carefully layer 10 mL of a density gradient medium (e.g., Lymphoprep, Table 2) to the bottom of the tube using a 10 mL pipette.
3. Centrifuge the tube at 800 g for 20 min with the brakes off. The PBMCs will now be visible as a white layer on top of the density gradient medium (Fig. 1b, left panel).
4. Use a Pasteur pipette to transfer the PBMCs into two new 50 mL tubes. Wash the cells twice with PBS.
5. Centrifuge the cells at 300 g for 15 min.
6. NOTE: Steps 6–8 are optional.
  Discard the supernatant and resuspend the pellet in 5 mL 1 x RBC lysis buffer (Table 2). Leave the cell solution for 10 min at RT
7. Stop the reaction by diluting the cells with 25 mL PBS.
8. Count the cells using the preferred method.
9. Centrifuge at 300 g for 15 min.
10. Discard the supernatant and resuspend the cells in FBS supplemented with 10% DMSO, aliquot, and store in liquid nitrogen.

CLL cells undergo rapid, spontaneous apoptosis outside of their natural microenvironment. To ensure that the monitored cell death in drug sensitivity screens is induced by drug treatment only, it is necessary to prevent the spontaneous apoptosis. This can be done by mimicking the tumor microenvironment by including microenvironmental stimuli in the growth conditions of the CLL cells. Below is a protocol that uses transient stimulation of the CLL cells by co-culturing them with fibroblasts that express APRIL, BAFF, and CD40L. The CLL cells are separated from the fibroblasts before the drug sensitivity screening so that the cell viability measurements only come from the CLL cells. This protocol has been used to predict treatment vulnerabilities in CLL, and to guide precision medicine for patients with relapsed disease [6, 8, 11]. The development of the co-culture protocol was previously reported [8].

#### Co-culture of CLL cells with APRIL/BAFF/CD40L+ fibroblasts (Day 1)

NOTE: Three 3T3 fibroblast cell lines stably expressing GFP-APRIL, GFP-BAFF, or GFP-APRIL + CD40L (CD40LA) are used in this protocol. The cell lines are cultured in separate cell culture flasks with RPMI 1640 medium (Table 2) supplemented with 1× sodium pyruvate, 1× MEM non-essential amino acids, penicillin/streptomycin, and 10% FBS (complete medium; Fig. 1b, second panel).

1. Detach the fibroblast cell lines from their culturing flasks by trypsinization. Transfer each cell line to a separate 15 mL tube.
2. Centrifuge the fibroblasts at 300 g for 5 min. Discard the supernatant.
3. Resuspend the fibroblasts in 5 mL complete medium. Keep the cells on ice to prevent them from re-attaching to the plastic.
4. Irradiate the APRIL+ and BAFF+ fibroblast cell lines at 125 Gy, and the CD40LA cell line at 50 Gy.
5. Seed the irradiated APRIL/BAFF/CD40L+ fibroblasts (1:1:1) in a tissue culture flask to allow a final ratio of 1:10 fibroblasts:CLL cells. Leave the flask in an incubator at 5% CO_2_, 37 °C to let the cells attach to the plastic.
6. Quickly thaw a frozen aliquot of CLL cells in a 37 °C water bath.
7. Wash the cells once with 10 mL complete medium.
8. Centrifuge at 300 g for 5 min. Discard the supernatant.
9. Resuspend the CLL cells in complete medium and transfer them to the culture flask containing APRIL/BAFF/CD40L+ fibroblasts.
10. Leave the co-culture in an incubator at 5% CO_2_, 37 °C for 24 h.

Drug sensitivity screens can be performed in different formats. Here, we describe a protocol for drug sensitivity screening in a 384-well plate format.

#### Drug sensitivity testing in a 384-well plate format (Day 2)

1. Separate the soluble CLL cells from the adherent fibroblast layer by transferring the cell culture medium to a 50 mL tube. Carefully rinse the flask with a 10 mL pipette to collect most of the CLL cells while leaving the fibroblasts undisturbed.
2. Filter the cell suspension using a 40 µm cell strainer to assure a single-cell suspension (Box 2, 2a).
3. Count the cells using the preferred method.
4. Centrifuge the cells at 300 g for 5 min. Discard the supernatant.
5. Resuspend the cells in complete medium to a final concentration of 2.0 × 10^5^–4.0 × 10^5^ cells/mL. This will allow for 5000–10,000 cells/well in a volume of 25 μL/well.
6. Transfer the cell suspension to preprinted 384-well drug plates at 25 μL/well using a liquid dispenser such as the CERTUS Flex (Fig. 1b, third panel). It is recommended to sonicate the valves of the dispenser before each use (Box 2, 1c).
7. Leave the plates in an incubator at 5% CO_2_, 37 °C for 72 h.

#### Measurement of cell viability with CellTiter-Glo (Day 5)

1. Add 25 μL pre-filtered CellTiter-Glo (Table 2) to each well in the 384-well plates (see Box 3 for experimental details and an alternative method).
2. Read the luminescence with a luminometer (Fig. 1b, fourth panel).

### Primary MM cells

MM cells cannot be biobanked but need to be processed fresh. The below protocol for isolation, culturing and drug sensitivity screening of MM cells was previously reported [23].

#### Isolation of bone marrow mononuclear cells (BMMCs) from MM patient BM samples (Day 1)

NOTE: Sections “Introduction”, “Drug sensitivity screening protocols”, “Drug library design” and “Reproducibility and quality controls of drug screens” should be performed under sterile conditions in a tissue culture hood. BM samples procured from MM patients should be processed the same day.

1. Pipette the BM gently up and down with a 10 mL pipette to remove clumps and filter the sample through a sterile 70 μm nylon filter into a 50 mL tube. Wash the filter once with 5 mL PBS.
2. Dilute the BM 1:1 with PBS.
3. Split the cell suspension equally into two 50 mL tubes.
4. Carefully layer 10 mL density gradient medium (Lymphoprep; Table 2) to the bottom of the tubes using a 10 mL pipette.
5. Centrifuge for 25 min at 800 g at room temperature, without breaks. The BMMCs are now visible on top of the density gradient medium layer (Fig. 1b, left panel).
6. Transfer the cells into two new 50 mL tubes using a Pasteur pipette.
7. Wash the cells with PBS by filling up the tube to 40–45 mL.
8. Centrifuge at 300 g for 15 min.
9. Discard the supernatant and wash the cells with PBS by filling up the tube to 40–45 mL.
10. Centrifuge at 300 g for 10 min.
11. Discard the supernatant and resuspend the cell pellet in PBS.

#### Removal of CD8^+^ T cells (Day 1)

After BMMC isolation, CD8^+^ cells are removed by addition of CD8 magnetic beads coated with anti-CD8 antibody (Dynabeads #11147D, Thermo Fisher Scientific) following the manufacturer’s protocol.

1. Count the BMMCs using the preferred method.
2. Centrifuge the BMMCs at 300 g for 5 min.
3. Resuspend the pellet in MACS buffer (1 mL per 1 × 10^7^ cells) and incubate with Dynabeads CD8 (25 μL per 1 × 10^7^ cells) for 30 min at 2–8 °C in the dark with gentle rotation.
4. Place the tube in a magnetic rack for 1–2 min to remove bead-bound CD8^+^ cells.
5. Transfer the supernatant to a new tube.
6. Centrifuge the cells at 300 g for 5 min.
7. Resuspend the pellet to a final concentration of 0.5–1 × 10^6^ cells/mL with RPMI 1640 medium (Table 2) supplemented with 2 mm L-glutamine, 10% fetal bovine serum, 1 µm sodium pyruvate, 1% penicillin and streptomycin (hereafter referred to as RPMI).

#### Stimulation of MM cells (Day 1)

Following isolation, the MM cells are stimulated with a T-cell expansion cocktail.

1. Culture the CD8-depleted BMMCs (0.5–1 × 10^6^ cells/mL) for 48 h at 37 °C in RPMI supplemented with human rIL-2 (100 U/mL) and human T cell activator CD3/CD28 magnetic beads (25 μL per 1 × 10^6 ^T cells, Dynabeads #11132D) according to the manufacturer’s instructions (Fig. 1b, second panel).

#### CD138^+^ MM cell enrichment (Day 3)

After stimulation, MM cells are enriched using CD138-MACS magnetic beads (Miltenyi Biotec, #130-051-301) according to the manufacturer’s instructions.

1. Transfer the cells to a tube and place it in a magnetic rack for 1–2 min to remove bead-bound T cells.
2. Transfer the supernatant to a new tube.
3. Count the cells using the preferred method.
4. Centrifuge the cells at 300 g for 5 min.
5. Resuspend the cell pellet in MACS buffer (80 μL per 2 × 10^7^ total cells) with CD138-MACS magnetic beads (20 μL per 2 × 10^7^ total cells) and incubate for 15 min at 2–8 °C in the dark with gentle rotation.
6. Place an LS MACS column (Miltenyi Biotec #130-042-401) onto a magnetic rack and wash once by adding 3 mL MACS buffer according to protocol. Let the MACS buffer run through.
7. Place a tube below the empty LS column.
8. Transfer the cell suspension to the LS column. Collect the run-through in the tube.
9. Wash the LS column three times with 1 mL MACS buffer. Collect the run-through in the same tube (CD138^−^ cells).
10. Replace the collection tube with a new tube.
11. Add 5 mL MACS buffer to the LS column. Flush out the bead-bound CD138^+^ MM cells by pushing a plunger into the column.
12. Centrifuge the collected cells at 300 g for 5 min.
13. Resuspend the cell pellet in 1 mL RPMI.
14. Count the cells using the preferred method.

#### Dispensing of cells into assay plates (Day 3)

1. Resuspend the cells in RPMI to a final concentration of 2 × 10^5^ cells/mL. This will allow for 5000 cells/well in a volume of 25 µL/well.
2. Transfer the cell suspension to preprinted 384-well drug plates at 25 µL/well using a liquid dispenser such as the CERTUS Flex (Fig. 1b, third panel). It is recommended to sonicate the valves of the dispenser before each use (Box 2, 1c).
3. Leave the plates in an incubator at 5% CO_2_, 37 °C for 72 h.

#### Measurement of cell viability with CellTiter-Glo (Day 6)

1. Add 25 μL pre-filtered CellTiter-Glo (Table 2) to each well in the 384-well plates (see Box 3 for experimental details and an alternative method).
2. Read the luminescence with a luminometer (Fig. 1b, fourth panel).

## Drug library design

### Single-drug dose-response curves

Drug sensitivity testing can be performed with pre-designed drug libraries that are commercially available from suppliers such as Selleck Chemicals (https://www.selleckchem.com/screening-libraries.html) or MedChemExpress (https://www.medchemexpress.com/), or with custom drug libraries designed by the user. The drug library is designed with high throughput in mind, accounting for the solubility of the compounds, favoring those that can be dissolved in DMSO or water. Each drug is typically tested at multiple concentrations, such as five 10-fold serial dilutions. The concentration range can be determined for each drug individually, or a more general approach can be applied where an equally broad concentration range is used for all drugs. The drug sensitivity curves provide information on drug efficacy and allow for calculation of parameters such as the EC50 (half maximal effective concentration), the bend points where the effect of the drug changes from exponential to asymptotic, and the dynamic range or the range located between the EC10 and EC90 (Fig. 1c). Fitting concentration-response curves to efficiently calculate EC50 values requires a minimum of 5 doses, with 6 being optimal: two doses before the low bend point, two doses after the high bend point and at least one dose, ideally two, in the slope (Fig. 1c) [31].

### Drug combinations

Different approaches can be taken when screening for drug interactions. The most complete approach would be to test the two drugs at every possible concentration combination, the so-called full matrix (Fig. 1d, left panel). However, this approach is highly resource-demanding. To reduce the number of patient cells and amount of reagents required for testing drug responses in multiple concentration combinations, the user can choose to test only part of the concentration-combinations using one of the following designs (Fig. 1d): (i) Fixed-concentration, or anchored, design: various concentrations of one agent are tested with a pre-defined concentration of the second agent (Fig. 1d, second panel); (ii) Fixed-ratio diagonal design: the diagonal dose-combination elements of the full pairwise concentration response matrix are tested (Fig. 1d, third panel); (iii) Fixed-ratio x-design: both diagonal concentration-combination elements (the “x”) of the full pairwise concentration response matrix are tested (Fig. 1d, right panel). Data analysis tools are available that can predict the full matrix of drug responses based on these fixed designs [32].

The definition of a concentration range for the single drug sensitivity curves provides information on the drug concentrations that may be used for drug combination screening. Those concentrations are recommended to fall between the EC10 and the EC90, referred to here as the dynamic range (Fig. 1c). Going below the EC10 poses a risk of using a concentration with little effect at all, while going above the EC90 will leave little room to detect additional effects of the drug combination compared to either single agent alone.

## Reproducibility and quality controls of drug screens

**Fig. 2:**
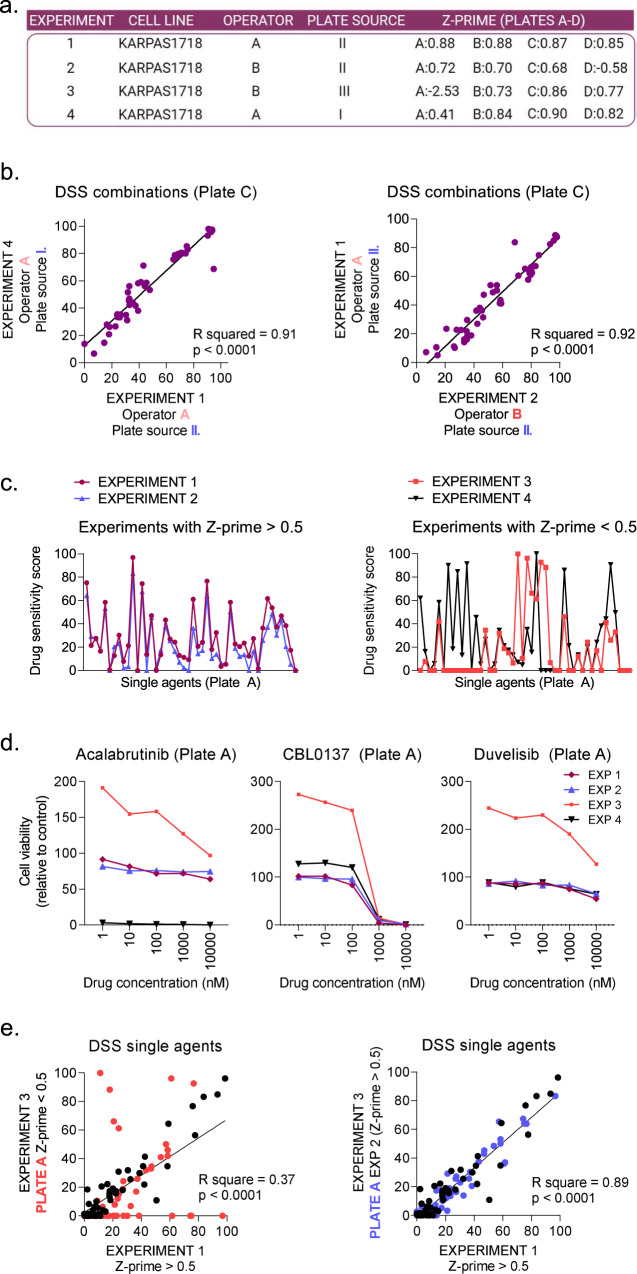
In vitro drug sensitivity is reproducible independently of operator and screening platform. KARPAS1718 cells were screened against a drug library consisting of 88 (NCMM and FIMM libraries) or 97 (DK-OPENSCREEN library) single agents and 95 (NCMM and FIMM libraries) or 102 (DK-OPENSCREEN library) drug combinations at five different concentrations. Cell viability was assessed with the CellTiter-Glo luminescent cell viability assay after 72 h. The drug sensitivity score (DSS) was calculated for each treatment based on the area under the dose-response curve. A high score indicates high sensitivity to the treatment. **a** Overview of the performed experiments. Experiment number (1–4), operator (A or B), plate source (I, NCMM; II, FIMM; III, DK-OPENSCREEN) and Z-prime on the four assay plates (A-D) are indicated. **b** Simple linear regression was performed for the responses to the combinations on plate C (Z-prime > 0.5) of the drug library, for the indicated experiments (1, 2, 4). Operator and plate source for each experiment are highlighted. **c** The DSS for single agents on Plate A are shown for experiments with Z-prime > 0.5 (1 and 2, left panel) and < 0.5 (3 and 4, right panel). **d** The cell viability relative to DMSO (0.1%) treated control cells are shown for KARPAS1718 cells treated with the indicated drugs. These agents were located on Plate A, which had a low Z-prime in experiment 3 (−2.53; coral curve) and experiment 4 (0.41; black curve). In experiments 1 and 2, the Z-prime for Plate A was > 0.5. **e** Simple linear regression was performed for the responses to the single agents of the drug library, for the indicated experiments (1, 2, 3). Responses to single agents on Plate A are highlighted in coral (left panel), while responses to single agents on plates B-D are shown in black. In the right panel, the responses to single agents on Plate A of experiment 3 are replaced by responses to the same agents from experiment 2 (blue data points).

Drug sensitivity screens have been shown to be reproducible over time [24]. Here, we further show that drug screens are reproducible independently of operator and what platform that printed the drug plates (the drug plate source; Fig. 2). Four experiments performed on the lymphoma cell line KARPAS1718 [33] and with variation in performance were selected for illustration purposes. The cell line was confirmed mycoplasma negative with the MycoAlert Detection Kit (Lonza, Basel, Switzerland). Two operators (A and B, Fig. 2a) performed the experiments, and the drug plates were obtained from three different sources (I-III, Fig. 2a). The drug sensitivity scores (DSS), which are quantitative measurements derived from the area under the concentration response curves with further normalization [34], were highly correlated between experiments performed by the same operator with different plate sources, or by different operators with the same plate source (R squared 0.91 and 0.92, respectively; Fig. 2b). These findings support the performance of one drug screen per patient sample and that the experiments can be performed by different users with drug plates produced by different sources without introduction of significant variability.

The Z-prime is commonly used as a quality control for drug screens [18]. This factor describes how well the positive and negative controls in the experiment separate and indicates the likelihood of false positives or negatives. Z-prime values between 0.5–1 are excellent, values between 0–0.5 may be acceptable, while values below 0 indicate a poor assay. In the experiments performed on the KARPAS1718 cell line, the drugs were distributed on four 384-well plates (A-D). In two of the experiments, the Z-prime for the A-plate was < 0.5 (0.41 and −2.53), while it was > 0.5 in the other two experiments (Fig. 2a). For the remaining plates, 11/12 (92%) of the experiments had a Z-prime > 0.5 (Fig. 2a). When the DSS for the drugs on Plate A were plotted for the four experiments, it showed that the two experiments with low Z-prime (Experiments 3 and 4) had many outliers relative to the high-quality experiments (Experiments 1 and 2; Fig. 2c). This trend was clearly visible when looking at the viability curves (Fig. 2d). Interestingly, the two experiments with Z-prime < 0.5 resulted in highly diverging results for many of the treatments (Fig. 2c, d). These findings highlight the importance of including quality controls in each experiment. Of note, we found that although part of an experiment (i.e. one of the plates) showed a Z-prime < 0.5, the other parts of the experiment with a Z-prime > 0.5 could still be used. In Fig. 2e, Plate A from Experiment 3 (Z-prime < 0.5) was replaced with Plate A from Experiment 2 (Z-prime > 0.5), resulting in a shift from low to high correlation with the DSS from Experiment 1 (Z-prime > 0.5; R squared 0.37 and 0.89, respectively). This indicates that it is acceptable to repeat only that part of the experiment which shows a Z-prime < 0.5, when relevant.

## Data analysis

Data analysis tools for drug screening data have been described in detail elsewhere [30, 35], and will not be discussed here.

## Discussion

High heterogeneity in clinicobiological features such as genetic aberrations and clinical outcomes among patients with hematologic cancers has led to the realization that treatment strategies tailored to the individual patient are needed to improve disease management. Genomic strategies do to some extent guide treatment decisions [36, 37], but further precision is required to avoid ineffective therapies [38].

Functional precision medicine guides treatment decisions based on functional analyses, often a drug sensitivity screen, of the patient’s tumor cells [15, 16]. This approach allows for the monitoring of treatment sensitivities in real time. The protocol is fast; it provides test results in 3–5 days after collection of the patient sample, with limited computational skills required for the data analysis. This is in contrast to genomic analyses which can take several weeks to process and require high computational skills for data analysis [16].

Here, we report our optimized and validated protocols for drug sensitivity screening of hematologic cancers. We show that reproducible results can be acquired on the same material analyzed by different operators or screened with drug libraries printed at different platforms. We previously reported that the results also are reproducible over time [24]. These features are critical for successful implementation in clinical trials and routine clinical practice. Clinical trials have been initiated that use these drug sensitivity screening results as biomarkers to guide precision medicine in hematologic cancers (Table 1), with encouraging results [4, 5, 19].

While we present ex vivo screening protocols that use the CellTiter-Glo assay or flow cytometry as a read-out, alternative approaches to ex vivo drug sensitivity screening have been established and previously reported, including three-dimensional cell culture models [39–43] and protocols with image-based read-outs [19, 44–46]. Continuous development of the screening protocols, including the consideration of microenvironmental effects on drug sensitivity, is expected to expand their applicability, both when it comes to disease indications and to drug classes that can be assessed. Miniaturization of the set-up further enables screening of cancers with very limited tumor material [47]. While functional precision medicine is spearheaded by hematologic cancers, development in solid tumors follows suit [15, 16]. This is a clear indication that functional precision medicine will find its place in future cancer care.

## Data Availability

The data that support the findings of this study are available from the corresponding author (sigrid.skanland@ous-research.no) upon reasonable request.
